# Site-Dependent Dynamic Life Cycle Assessment of Human Health Impacts from Industrial Air Pollutants: Inhalation Exposure to NO_x_, SO_2_, and PM_2.5_ in PVC Window Manufacturing

**DOI:** 10.3390/toxics14010023

**Published:** 2025-12-25

**Authors:** Patrice Megange, Amir-Ali Feiz, Pierre Ngae, Thien Phu Le, Patrick Rousseaux

**Affiliations:** 1 Le Laboratoire de Mécanique et d’Energétique d’Evry (LMEE), Université Évry Paris-Saclay, 91020 Evry, France; amirali.feiz@univ-evry.fr (A.-A.F.); pierre.ngae@univ-evry.fr (P.N.); thienphu.le@univ-evry.fr (T.P.L.); 2 Institut Pprime, Université de Poitiers, CNRS UPR 3346, IRIAF, 11 rue Archimède, 79000 Niort, France; patrick.rousseaux@univ-poitiers.fr

**Keywords:** industrial air emissions, human health risk, life cycle assessment (LCA), site-dependent dynamic LCA (SdDLCA), atmospheric dispersion modeling, PM_2.5_, NO_x_, SO_2_

## Abstract

Industrial air emissions are major contributors to human exposure to toxic pollutants, posing significant health risks. Life cycle assessment (LCA) is increasingly used to quantify human toxicity impacts from industrial processes. Conventional LCA often overlooks spatial and temporal variability, limiting its ability to capture actual inhaled doses and exposure-driven impacts. To address this, we developed a site-dependent dynamic LCA (SdDLCA) framework that integrates conventional LCA with Enhanced Structural Path Analysis (ESPA) and atmospheric dispersion modeling. Applied to the production of double-glazed PVC windows for a residential project, the framework generates high-resolution, site-specific emission inventories for three key pollutants: nitrogen oxides (NO_x_), sulfur dioxide (SO_2_), and fine particulate matter (PM_2.5_). Local concentration fields are compared with World Health Organization (WHO) air quality thresholds to identify hotspots and periods of elevated exposure. By coupling these fields with the ReCiPe 2016 endpoint methodology and localized demographic and meteorological data, SdDLCA quantifies human health impacts in Disability-Adjusted Life Years (DALYs), providing a direct measure of inhalation toxicity. This approach enhances LCA’s ability to capture exposure-driven effects, identifies populations at greatest risk, and offers a robust, evidence-based tool to guide industrial planning and operations that minimize health hazards from air emissions.

## 1. Introduction

Air pollution is recognized as one of the foremost environmental determinants of human health worldwide. The World Health Organization (WHO) identifies ambient air pollution as a major cause of premature mortality, with fine particulate matter (PM_2.5_), nitrogen dioxide (NO_2_), and sulfur dioxide (SO_2_) being linked to cardiovascular, respiratory, and systemic diseases, even at relatively low exposure levels [1]. In response to these health risks, several public health regulations, including the WHO Global Air Quality Guidelines, define concentration limits for key pollutants such as PM_2.5_, PM_10_, NO_x_ and SO_2_ based on 24 h averages [2]. These guidelines provide evidence-based thresholds to protect population health and serve as reference values for environmental policies and air quality management worldwide.

Recent epidemiological studies have highlighted those short-term peaks in PM_2.5_ and NO_2_ exposure can trigger measurable health outcomes. In particular, a multi-city analysis demonstrated that daily variations in these exposures significantly influence cardiovascular and respiratory mortality [3].

Among the various sources of air pollution, industrial emissions constitute a distinct and substantial contributor to population exposure [4]. Quantitative assessments indicate that combustion-related industrial sources contribute markedly to particulate-matter-attributable mortality across multiple cities worldwide [5]. Prospective atmospheric modeling has revealed that industrial and energy-related emissions strongly affect premature mortality under different emission scenarios, with considerable spatial variability. Incorporating high-resolution inventories, such as the HTAP_v3 emission mosaic, further improves the robustness and reliability of air quality and health impact assessments [6].

**Figure 1:**
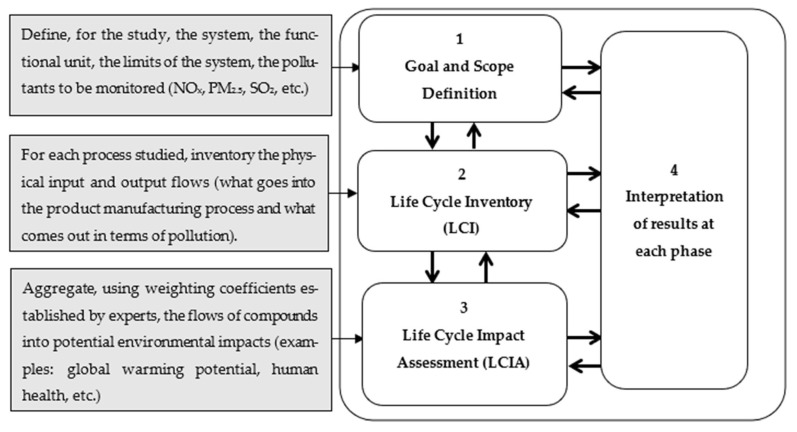
Four main phases of LCA, inspired by [11].

Therefore, accurately quantifying these toxicological impacts is critical for protecting human health and guiding effective mitigation strategies. In this context, life cycle assessment (LCA) has emerged as a widely recognized framework for evaluating the environmental impacts of products and industrial systems throughout their entire life cycle, including potential human health effects [7]. Its systemic perspective and ability to integrate multiple emission sources make life cycle assessment a valuable framework for assessing the health implications of industrial activities, particularly when combined with health risk assessment, as demonstrated in studies of industrial emission scenarios [8]. International standards formalizing the LCA methodology [9,10] define four main phases—illustrated in Figure 1: (1) goal and scope definition, (2) inventory analysis, (3) impact assessment, and (4) interpretation—enabling its widespread application in policy-making, eco-design, and sustainability assessments.

Neglecting temporal differentiation in life cycle inventories can bias impact estimates for global categories such as climate change [12]. Dynamic life cycle assessment approaches that explicitly account for temporal variation can reduce such biases and provide more accurate characterization of emissions over time [13]. By extension, this limitation is expected to be even more critical for exposure-driven categories, including human toxicity, which are inherently sensitive to emission timing and exposure dynamics. Moreover, insufficient spatial resolution can obscure local hotspots and misrepresent health impacts, particularly in densely populated industrial areas; studies integrating life cycle assessment with spatially resolved modeling and exposure assessment have highlighted the importance of considering local conditions to improve the accuracy of health impact evaluations [8].

Several methodological advances have sought to address the limitations of conventional LCA. Existing frameworks, such as PANGEA [14], Enhanced Structural Path Analysis (ESPA) [15], dynamic LCA more broadly [16,17], and ReCiPe [18], provide valuable temporal or regional insights, yet they rarely integrate high-resolution spatiotemporal variability necessary for applications such as policy-making, eco-labeling, and sustainable product design. These approaches also often rely on simplified assumptions regarding pollutant fate and human exposure and remain weakly coupled with high-resolution atmospheric dispersion models, limiting their ability to capture the combined influence of emission timing, local meteorology, topography, and population distribution on actual inhaled doses. Temporal resolution is essential for capturing emission peaks and inhaled doses [19], while spatial resolution accounts for local factors such as weather, topography, and population density that influence pollutant transport and exposure [20,21].

Given the demonstrated importance of short-term exposure peaks for health outcomes, the term “dynamic” in this context refers to the explicit temporal differentiation of emissions and exposure processes, which enables capturing these variations and emission peaks known to drive inhalation exposure. By incorporating emission timing and its interaction with local meteorology and population distribution, dynamic LCA frameworks provide a more realistic estimation of inhaled doses and human health impacts from industrial air pollution.

While life cycle inventories quantify pollutant emission rates, human exposure and associated health risks are governed by ambient concentrations resulting from atmospheric dispersion. Accordingly, this study adopts a concentration-based approach by coupling life cycle inventory data with site-specific dispersion models.

The objective of the present study is to develop a robust and flexible site-dependent dynamic life cycle assessment (SdDLCA) framework for assessing human health impacts from industrial air emissions. The proposed approach integrates temporal dynamics of technological and energy flows, pollutant transport, atmospheric dispersion, and population exposure, while remaining fully compatible with established LCA tools. Applied to a PVC window manufacturing for a residential project, the SdDLCA framework demonstrates how spatiotemporal differentiation can lead to more realistic estimates of human exposure and health damage, thereby supporting improved monitoring, management, and mitigation strategies for industrial air pollution.

## 2. Materials and Methods

### 2.1. Site-Dependent Dynamic Life Cycle Assessment Framework

The proposed framework builds upon the classical matrix formulation of the life cycle inventory [22]:

(1) $$g=B\times A^{-1}\times f$$

where g represents the inventory vector (kg) quantifying pollutant emissions and resource extractions, B is the environmental intervention matrix, A is the technological matrix defining product and material flows, and f is the reference flow vector describing the functional unit.

To integrate temporal and spatial disaggregation, this study applies the ESPA model. ESPA introduces time and location into LCA by assigning temporal distribution functions to each technological flow—quantity of substances transformed and exchanged between technological processes—and elementary flow—flow of matter or energy entering the system under study and drawn from the environment without prior human transformation, and leaving the system under study and being released into the environment without further human transformation. These functions describe how local industrial processes operate and how emissions evolve over time, replacing the conventional assumption of instantaneous flows. Temporal data such as manufacturing lead time, Takt time (the production rate required for on-time delivery), and the sequence of industrial operations are used to represent the dynamics of production and, consequently, those of pollutant emissions, thereby generating an evolving and spatially explicit life cycle inventory.

**Figure 2:**
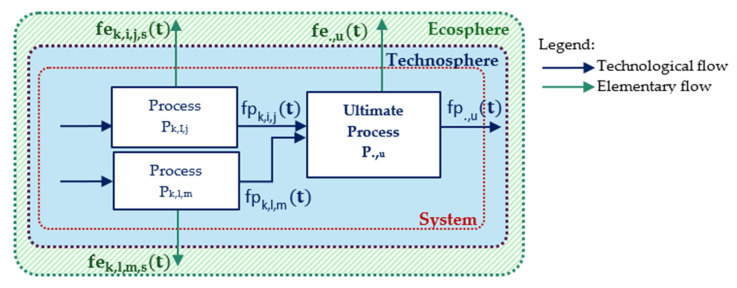
Example of a complex system.

The ESPA framework combines these temporal and spatial distributions through a convolution product between technological and elementary flows (mainly atmospheric pollutants in this study). This operation propagates information through both time and space in the life cycle inventory result. Given the complexity of this article’s analyzed system (Figure 2), the integration of temporality considerations relies on an adapted equation based on previous work [23]:

(2) $$g_{k,\;s}\left(t\right)=\sum_{i}\sum_{j}\left[\left(r_{i,j}(t)*{fp}_{k,i,j}(t)\right)*{\mathrm{fp}}_{u}\left(t\right)\right]*{\mathrm{fe}}_{k,i,j,s}\left(t\right)+\sum_{l}\sum_{m}\left[\left(r_{l,m}(t)*{fp}_{k,l,m}(t)\right)*{\mathrm{fp}}_{u}\left(t\right)\right]*{\mathrm{fe}}_{k,l,m,s}\left(t\right)+\ldots$$

where $g_{k,\;s}\left(t\right)$ (kg) is the spatialized dynamic inventory of the pollutant “s” emitted at site “k”, ${\mathrm{fp}}_{.,u}\left(t\right)$ ((per functional unit) is the spatiotemporal technological function of the ultimate process P_u_, ${\mathrm{fe}}_{.,u}\left(t\right)$ (kg) is the spatiotemporal unitary flow function of the air pollutant “s” emitted in the ecosphere by the ultimate process P_.,u_ in a potential other localization “.”, ${\mathrm{fp}}_{k,i,j}\left(t\right)$ and ${\mathrm{fp}}_{k,l,m}\left(t\right)$ (kg/(unit of the Functional Unit)) are spatially and temporally differentiated components of the technological matrix A and “∗” denotes the convolution product [24].

### 2.2. Coupling ESPA with Numerical Atmospheric Dispersion Models

To translate temporal emission flows into environmental concentrations, pollutant emissions were coupled with numerical atmospheric dispersion models. The choice of model depends on terrain complexity.

#### 2.2.1. Gaussian Plume Model

For flat terrain, the Gaussian plume model estimates pollutant concentrations near emission sources:

(3) $$C_{s}\left(x,y,z,t\right)=\frac{Q_{k,s}(t)}{2\pi U_{k}\sigma_{y}(x)\sigma_{z}(x)}\exp(\left(-\frac{{\left(y-y_{0}\right)}^{2}}{2\sigma_{y}^{2}(x)}\right)\left[\exp\left(-\frac{{\left(z-H\right)}^{2}}{2\sigma_{z}^{2}(x)}\right)+\alpha \exp\left(-\frac{{\left(z+H\right)}^{2}}{2\sigma_{z}^{2}(x)}\right)\right]$$

where $C_{s}\left(x,y,z,t\right)$ (kg/m^3^) is the concentration of pollutant “s”, $Q_{k,s}(t)$ (kg/s) represents the mass flow rate of substance “s” emitted by the considered process near the site “k”, $U_{k}$ (m/s) is local wind speed, $\sigma_{y}(x)\;and\;\sigma_{z}(x)$ (meter) are dispersion coefficients, determined using Briggs’ empirical dispersion coefficients [25], H (meter) is the effective stack height, and α is the terrain reflection coefficient.

(4) $$Q_{k,s}(t)=\frac{g_{k,s}\left(t\right)}{∆t}$$

where Δt (second) is the chosen time step.

#### 2.2.2. CALPUFF Model

For complex or hilly terrain, the CALPUFF 7.3.1 software simulates pollutant dispersion as discrete “puffs” over time [26]. Concentrations are calculated by summing contributions from all puffs using an analogous formulation to the Gaussian plume, with puff-specific temporal resolution ${∆t}_{\mathrm{puff}}$ and number of puffs $N_{\mathrm{puff}}.$

(5) $$C_{s}\left(x,y,z,t\right)=\sum_{i=1}^{N_{puff}}\frac{Q_{s}\times ∆t_{puff}}{2\pi^{3/2}\sigma_{x}^{i}\sigma_{y}^{i}\sigma_{z}^{i}}e^{\left(-\frac{{\left(x-x_{0}\right)}^{2}}{2{\left(\sigma_{x}^{i}\right)}^{2}}\right)}e^{\left(-\frac{{\left(y-y_{0}\right)}^{2}}{2{\left(\sigma_{y}^{i}\right)}^{2}}\right)}\left[e^{\left(-\frac{{\left(z-H\right)}^{2}}{2{\left(\sigma_{z}^{i}\right)}^{2}}\right)}+{\alpha e}^{\left(-\frac{{\left(z+H\right)}^{2}}{2{\left(\sigma_{z}^{i}\right)}^{2}}\right)}\right]$$

This hybrid approach produces spatiotemporally resolved pollutant concentrations, capturing both emission timing and terrain/meteorology effects.

### 2.3. Human Health Impact Assessment

Pollutant concentrations were translated into human health impacts using the ReCiPe 2016 endpoint methodology, expressing damage in Disability-Adjusted Life Years (DALYs). The model follows a four-step causal chain:

1. The fate factor of substance “s” (${FF}_{s}$) traces pollutant dispersion, accounting for local terrain, meteorology, and residence time;
2. The intake fraction by inhalation (${IF}_{inh}$) relates environmental concentration to population dose;
3. The Effect Factor of substance “s” $({EF}_{s}$) converts dose to incidence of health effects;
4. The Damage Factor (DF) converts cases into DALY.

The spatiotemporal Human Health Characterization Factor is given by:

(6) $${CF}_{HH,s}(t)={FF}_{s}(t)\times {IF}_{inh}\times {EF}_{s}\times DF$$

and the absolute damage to human health is:

(7) $$D_{\mathrm{HH}}(t)=\sum_{s}{CF}_{HH,s}\times g_{k,s}\left(t\right)$$

In this study, the key factors in the causal chain are adapted to capture the spatial and temporal variability inherent to the life cycle of the analyzed system or product. By coupling numerical atmospheric dispersion results with the ReCiPe 2016 characterization framework, a more accurate representation of human exposure is achieved, explicitly accounting for local meteorology, topography, and population distribution. The subsequent subsections describe the computation of each element contributing to the human health characterization factor.

#### 2.3.1. Temporal Fate Factor ${FF}_{s}(t)$ of a Substance “s”

In order to address the area of improvement previously mentioned, the secondary coupling employs the Gaussian plume model or the puff model to integrate local and dynamic parameters—such as stack height, climatic variability, wind behavior, and topography—into the computation of the spatiotemporal fate factor FF_s_(t). This formulation is primarily derived from the Pennington equation [27]:

(8) $$M_{s}=FF_{s}\times Q_{s}$$

where $M_{s}$ (kg_intake_) is the vector having as components the masses of the pollutant accumulated in each exposed area and likely to be inhaled by the affected population. $Q_{s}$ (kg_emitted_/day) is the vector having as its components the continuous emission rates of the pollutant “s” in this same compartment and $FF_{s}$ the fate factor of the substance “s”.

Consequently, as part of this research work, the calculation of the fate factor is carried out with the equation:

(9) $${\mathrm{FF}}_{s}(t)=\frac{\sum C_{s}(x,y,z)\times V}{Q_{k,s}(t)}$$

where ${\mathrm{FF}}_{s}\left(t\right)$ (kg_intake_/(kg_emitted_/day)) quantifies, with a spatiotemporal distribution, how contaminants are dispersed in the environment while integrating the realistic evolution of the substance emitted in the contaminated compartment. It is also defined as the specific residence time of an emitted chemical substance. $Q_{k,s}(t)$ (kg_emitted_/day) is the mass flow rate of substance “s” determined with Equation (4) and $C_{s}\left(x,y,z,t\right)$ (kg_intake_/m^3^) is its concentration calculated with Equations (3) or (5). V (m^3^) is the volume of the affected tropospheric zone. To determine volume V, the calculation is based on the surface area of the affected zone and the selected tropospheric height, which extends from the ground up to approximately 2 m above the surface—corresponding to the average human breathing height. This layer lies within the surface boundary layer, where pollutant concentrations most directly influence human health. The sigma symbol (∑) indicates that a compilation of different gridded concentrations in the neighboring affected area is carried out from the daily local concentration results of the studied pollutants (SO_2_, NO_x_ and PM_2.5_).

#### 2.3.2. Intake Fraction ${IF}_{inh}$

The ${IF}_{inh}$, is determined with the following equation:

(10) $${IF}_{inh}=\frac{{\mathrm{IR}}_{\mathrm{inh}}\times N}{V}$$

where ${\mathrm{IR}}_{\mathrm{inh}}$ (m^3^/person/time unit) is the rate of individual human air consumption per inhalation. It will be adapted to the temporal scale of the scenario by using the temporal unit used in the context of the study. The daily average of ${\mathrm{IR}}_{\mathrm{inh}}$ is estimated at 13 m^3^/person/day for an individual with an average weight of 70 kg. N represents the number of inhabitants in the studied and exposed area and V (m^3^) the volume of air in the affected tropospheric zone. ${IF}_{inh}$ is unitless (kg_inh_/kg_emitted_).

#### 2.3.3. Effect Factor ${EF}_{s}$ of a Substance “s” and the Damage Factor DF

Sulfur dioxide (SO_2_) and nitrogen oxides (NO_x_) do not directly affect human health through inhalation but act as precursors to secondary particulate matter (PM_2.5_). Consequently, ReCiPe 2016 does not provide specific Effect Factors (EFₛ) for these substances. Instead, their impacts are estimated using Particulate Matter Formation Potentials (PMFPₛ), which quantify the mass of PM_2.5_ generated per kilogram of precursor emitted.

The corresponding Effect Factor for each precursor is then derived using the following relationship:

(11) $${EF}_{s}={PMFP}_{s}\times {CF}_{{PM}_{2.5}}$$

where ${CF}_{{PM}_{2.5}}$ is the conversion factor PM_2.5_ → endpoint (DALY/kg PM_2.5_).

The ${PMFP}_{s}$ and ${CF}_{{PM}_{2.5}}$ values, according to the studied pollutants, are shown in Table 1.

The exact DF used for each individual case is not published separately; it is integrated into ${CF}_{{PM}_{2.5}}$.

#### 2.3.4. Method Overview

**Figure 3:**
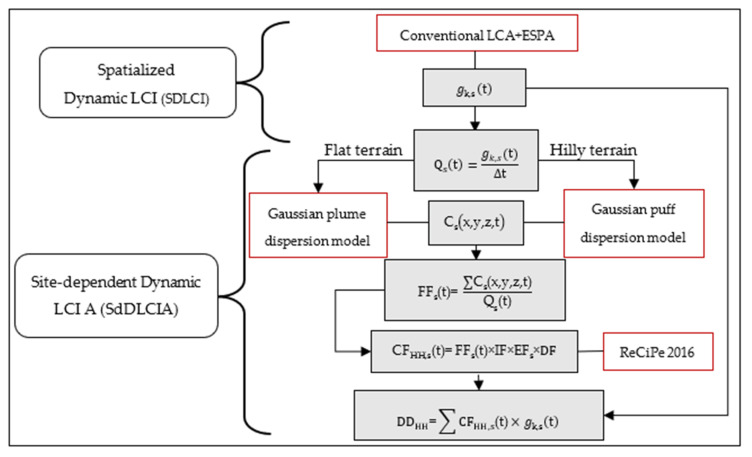
Algorithm of SdDLCA model.

All previous relationships are then incorporated into Equation 7 to quantify the impact on human health. In this way, the SdDLCA model improves the spatial and temporal resolution of impact assessments associated with atmospheric emissions. Our methodology follows a structured sequence of phases, each building on the previous to ensure a coherent and consistent analysis. Figure 3 below illustrates the overall algorithm and the interactions between the different phases.

### 2.4. Temporal Scale Selection

The selection of temporal resolution is a key methodological parameter in dynamic LCA, particularly for impact categories sensitive to short-term variations, such as human health. In the first stage of this study—the SDLCI—an hourly time step was adopted to better capture the temporal dynamics of industrial processes. This level of resolution reflects the way operational data are typically collected in industrial contexts, where machine time, energy use [30], and production cycles are often measured and recorded on an hourly basis. Such granularity ensures that the SDLCI faithfully represents process variability and emission fluctuations throughout the operating period. In the subsequent stages, namely the pollutant dispersion modeling and the SdDLCIA, a daily time step was employed for both concentration estimation and final impact calculation. This decision is supported by three main considerations.

1. Although the SDLCI is based on an hourly time step, adopting a daily resolution in the subsequent assessment stages remains consistent with the operational reality of industrial and supply-chain management, where key activities—including transport, delivery scheduling, and energy monitoring—are generally planned and tracked on a day-to-day basis [31]. This dual-scale approach maintains operational relevance while managing computational feasibility.
2. A daily resolution is essential for ensuring alignment with environmental health benchmarks and atmospheric dispersion modeling practices. Several tools used for exposure assessment, including atmospheric dispersion models, typically operate with daily or sub-daily time steps [32]. This temporal consistency allows for the accurate representation of pollutant dispersion and population exposure on a daily scale, ensuring that results are compatible with regulatory frameworks. The integration of dynamic life cycle assessment (LCA) outputs with pollutant dispersion simulations and exposure-based impact characterization thus becomes feasible, as daily averages provide a coherent time scale for assessing both long-term health risks and short-term exposure fluctuations. Consequently, this daily resolution facilitates the effective linking of modeling efforts with established health guidelines, supporting more precise evaluations of the health impacts of industrial air emissions.
3. This resolution allows the model to account for temporal variability in emissions and exposure induced by meteorological changes or operational fluctuations. Prior studies have shown that temporal resolution can substantially influence LCA results for human toxicity and ecotoxicity impact categories, with sub-daily or daily time steps providing improved accuracy compared to aggregated annual data [12,33].

In this work, emissions are modeled at a daily time step and characterized using the ReCiPe 2016 method at the Human Health endpoint level. The resulting daily impact scores are then aggregated at seasonal and annual scales to capture meteorological effects and ensure comparability with conventional static LCAs. Although ReCiPe characterization factors are not inherently time-dependent, their application to temporally resolved inventories enables the detection of short-term variations and critical emission periods, thus enhancing interpretative value.

Overall, adopting an hourly time scale at the inventory stage and a daily one for impact assessment provides a balanced compromise between methodological rigor, operational realism, and regulatory consistency. This multi-scale approach ensures that the SdDLCA framework accurately captures the temporal variability of industrial emissions while remaining fully compatible with conventional ReCiPe-based impact assessment methods.

### 2.5. Case Study Contextualization

The feasibility study evaluates the potential human health impacts associated with the manufacturing of double-glazed PVC windows covering a total surface area of 497 m^2^ for a residential building. The analysis was conducted in accordance with the methodological framework defined by ISO 14040 [9] and ISO 14044 [10].

Only the main air pollutants identified by the World Health Organization as most harmful to human health were considered, namely nitrogen oxides (NOx, including NO and NO_2_), fine particulate matter (PM_2.5_), and sulfur dioxide (SO_2_).

The LCA was performed using OpenLCA 2.4 software in combination with the Ecoinvent v3.10 database [34]. Algebraic and numerical computations were carried out using MATLAB R2024b and Microsoft Excel.

**Figure 4:**
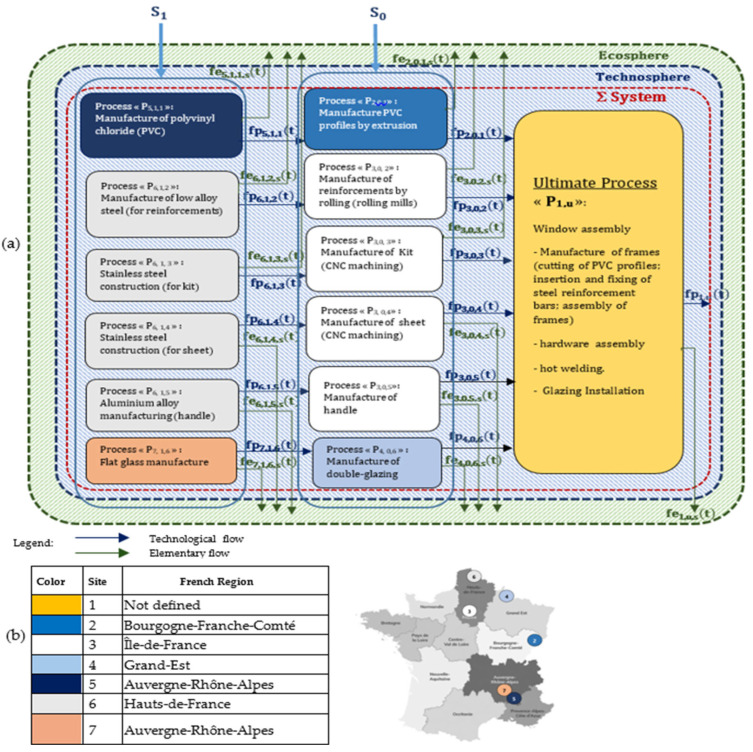
(**a**) Diagram of the complex studied system and (**b**) location of the French region of each studied process.

A spatio-temporal life cycle assessment (LCA) was carried out, distinguishing foreground processes from background processes. Figure 4 illustrates in detail the complex system studied, showing its industrial sites distributed across French regions.

**Figure 5:**
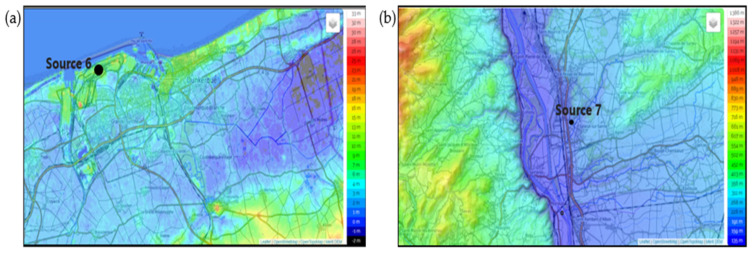
Topographic maps of (**a**) site 6 and (**b**) site 7. Source 6 and Source 7 indicate the locations of industrial air emissions.

To contextualize the case study and illustrate the Site-dependent Dynamic LCA approach, two industrial sites with contrasting environmental conditions were selected: Site 6 and Site 7, whose topographic maps are shown in Figure 5a,b.

**Figure 6:**
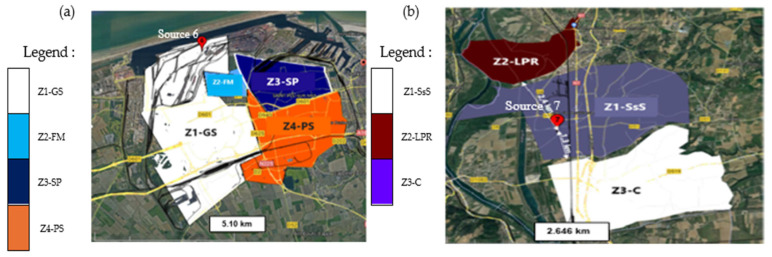
Surrounding zones of (**a**) site 6 and its neighboring towns, where Source 6 denotes the location of its industrial air emissions, and (**b**) site 7 and its neighboring towns, where Source 7 denotes the location of its industrial air emissions.

Site 6, located in a flat, low-relief area, represents a context of relatively simple pollutant dispersion. Four neighboring zones were delineated according to their proximity to the emission source 6: Z1-GS (Grande-Synthe), Z2-FM (Fort-Mardyck), Z3-SP (Saint-Pol), and Z4-PS (Petite-Synthe), as illustrated in Figure 6a. In contrast, Site 7, situated in a region with complex terrain characterized by plateaus and medium-altitude mountains, highlights the influence of topography on atmospheric dispersion. Three surrounding zones were defined according to their proximity to the emission source 7: Z1-SsS (Salaise-sur-Sanne), Z2-LPR (Le-Péage-Roussillon), and Z3-C (Chanas), as shown in Figure 6b.

These two sites and their site-specific delineations enable the framework to account for spatial variability in environmental exposure, integrating differences in local meteorology, land use, and population distribution. Such consideration ensures that the environmental impacts assessed through the Site-dependent Dynamic LCA reflect realistic, location-specific conditions.

The temporal sequencing of each process was defined to meet the requirements of the functional unit under realistic production conditions. Complementary data collection enabled the definition of an “absolute zero” reference date, corresponding to the completion of the final assembly process (P_1,u_) for the 497 m^2^ of windows.

The functional unit was defined as follows:

“to close a set of permanent openings totaling 497 m^2^ in an exterior wall, ensuring light transmission, manual operation, thermal insulation, watertightness, wind resistance, air permeability, and sound insulation, in accordance with the RE2020 regulation [35] and best practices over a 50-year lifespan.”

## 3. Results and Discussion

This section presents the results of the Site-dependent Dynamic Life Cycle Assessment (SdDLCA) framework, highlighting how high-resolution spatial and temporal differentiation improves the evaluation of human health impacts from industrial air emissions. The analysis focuses on the production of 497 m^2^ of double-glazed PVC windows for a residential project. Beyond numerical results, the SdDLCA framework enables a progressive, exposure-oriented assessment rarely addressed in existing dynamic LCA studies. While most dynamic LCA approaches focus on global impacts such as climate change, this work explicitly targets local human health effects. By coupling with atmospheric dispersion models, the assessment moves from emission rates to ambient concentrations, providing a realistic representation of exposure conditions. Although endpoint indicators are less robust than midpoint metrics, the resulting health damage estimates offer a clear and communicative portrayal of local environmental impacts.

### 3.1. Spatialized Dynamic Life Cycle Inventory (SDLCI)

**Figure 7:**
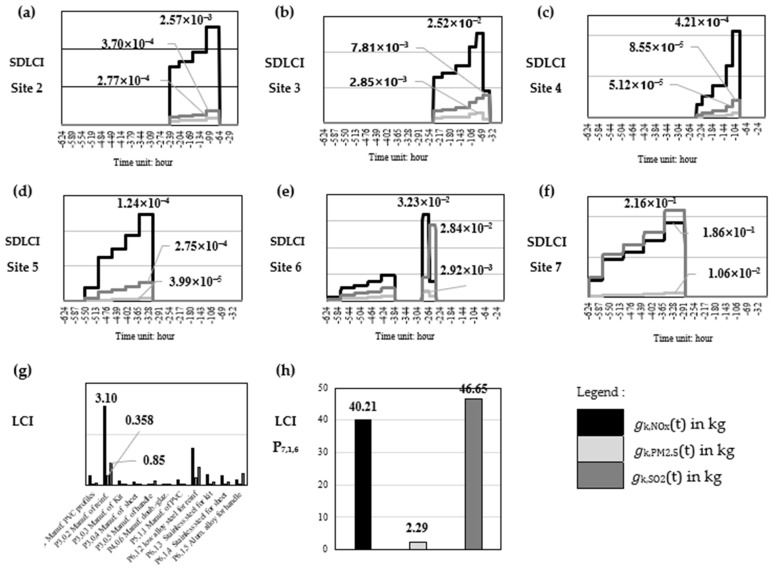
(**a**–**f**) illustrates the temporal evolution of NO_x_, SO_2_ and PM_2.5_ emissions from sites 2 determined by the spatially distributed dynamic life cycle inventory (SDLCI) approach; (**g**) presents the results of the conventional LCI for all processes, excluding P_7,1,6_; (**h**) presents the result of the conventional LCI only for the P_7,1,6_ process.

The dynamic behavior of manufacturing processes was noted to complement the Ecoinvent database. The SDLCI was established using Equation (2), integrating spatiotemporal disaggregation of each manufacturing process. Figure 7a–f shows the temporal evolution of emissions for each site and pollutant, while Figure 7g,h present conventional LCI results for comparison. The SDLCI was established using Equation (2), integrating the spatiotemporal disaggregation of each process.

Figure 7a–f shows the temporal evolution of emissions for each site and pollutant, while Figure 7g,h presents the conventional LCI for comparison.

Peak emissions occur during process P_7,1,6_ at Site 7, with elevated emissions also observed at Site 6. This is consistent with existing knowledge: metallurgical operations (Site 6) and flat-glass production (Site 7) are well-known sources of substantial NO_x_, SO_2_, and particulate emissions [36,37].

The SDLCI preserves the overall mass balance while capturing site-dependent heterogeneity and temporal variability. This highlights the added value of dynamic inventories: conventional LCI obscures emission peaks and temporal patterns, potentially biasing exposure assessment.

This aligns with earlier work in dynamic LCI, which has shown that static inventories tend to distort peak emission magnitudes and misrepresent exposure-determining emission timing [18,33]. Our results reinforce these conclusions by demonstrating that spatiotemporal differentiation significantly reduces inventory bias without altering total emitted mass.

### 3.2. Pollutant Concentrations

Atmospheric dispersion was simulated using ESPA–Gaussian/CALPUFF coupling. This step translates emissions into concentrations, capturing the combined effects of meteorology, topography, and source–receptor geometry.

#### 3.2.1. Site 6 (Hauts-de-France Region)

**Figure 8:**
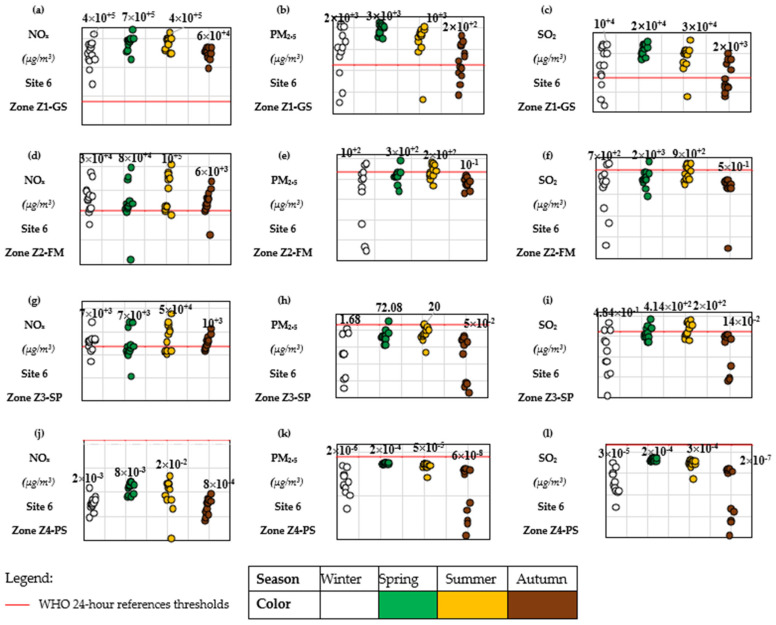
Seasonal mean daily potential concentrations (2020 weather conditions) of NO_x_, PM_2.5_, and SO_2_ (µg/m^3^) in the four study areas surrounding Site 6, calculated using the ESPA model coupled with the GAUSSIAN atmospheric dispersion model. Figure (**a**–**c**) shows the seasonal distribution in zone Z1-GS, figure (**d**–**f**) in zone Z2-FM, figure (**g**–**i**) in zone Z3-SP, and figure (**j**–**l**) in zone Z4-PS. Each figure indicates the WHO 24 h limit values with a horizontal red line (25 µg/m^3^ NO_x_, 15 µg/m^3^ PM_2.5_, 40 µg/m^3^ SO_2_).

At Site 6, the concentrations of NO_x_, PM_2.5_, and SO_2_ obtained through the ESPA + Gaussian coupling (Figure 8a–l) reveal several exceedances of WHO threshold values. These findings are consistent with meteorological data from the 2020 Dunkerque forecast [38], which reported weak winds (<2.5 m/s) from the west, northeast, and north during critical periods. Such low wind speeds, combined with the proximity of the emission source (<2.5 km), favor pollutant accumulation in the sectors Z1-GS, Z2-FM, and Z3-SP, in contrast to the more distant Z4-PS area. In addition, the results show significant day-to-day and seasonal variability in concentrations, driven by emission height, atmospheric stability, and seasonal meteorology—all factors that justify and validate the use of coupled atmospheric dispersion models for this type of analysis.

This confirms the importance of coupling SDLCI with dispersion modeling: pollutants behave non-linearly in space and time, consistent with well-established atmospheric dispersion principles.

#### 3.2.2. Site 7 (Auvergne–Rhône-Alpes Region)

Site 7 presents a more complex topography, combining plateaus and mid-altitude mountain ranges. For this reason, the CALPUFF system—an advanced modeling framework suitable for complex terrain, variable weather conditions, and physicochemical transformations—was selected to simulate pollutant dispersion. However, the resulting concentrations at Site 7 remain consistently below WHO reference thresholds. Although these results are therefore not detailed in this article, they nonetheless provide a reassuring first environmental assessment of flat-glass manufacturing emissions and their limited potential impact on nearby populations. Site 7 thus serves as a contrasting case study illustrating how complex terrain and dispersion conditions can mitigate exposure despite substantial industrial emissions.

#### 3.2.3. General Trends and Key Observations

Across both study sites, several general trends emerge from the concentration-based modeling results. First, the analysis confirms that pollutant concentrations, rather than emission rates alone, constitute the most relevant indicator for exposure and health-related assessment, as they integrate the combined effects of atmospheric dispersion, local meteorological conditions, and site-specific characteristics.

At Site 6, moderate-to-high emission rates, combined with unfavorable dispersion conditions, lead to episodic exceedances of WHO guideline values for NO_x_, PM_2.5_, and SO_2_, primarily due to unfavorable dispersion conditions, low wind speeds, and the close proximity of emission sources. In contrast, at Site 7, despite the use of a more advanced dispersion model adapted to complex terrain, modeled concentrations remain systematically below reference thresholds. This highlights the dominant role of local meteorology, topography, and source–receptor geometry in shaping ambient concentration levels.

A key observation is therefore that industrial processes associated with relatively high pollutant emission rates may result in markedly different exposure patterns depending on regional and site-specific contexts. These results illustrate the non-linear relationship between emissions and concentrations and underscore the necessity of coupling life cycle inventory data with atmospheric dispersion modeling. Such an approach enables a more realistic, spatially and temporally resolved assessment of potential population exposure, which cannot be captured by emission-based indicators alone.

### 3.3. Site-Dependent Dynamic Life Cycle Impact Assessment (SdDLCIA)

Building on the concentration patterns and site contrasts identified in Section 3.2, the third coupling step—linking atmospheric dispersion outputs with ReCiPe 2016—provides the final spatiotemporal human health impact assessment. This stage translates the modeled concentration fields into exposure-driven health impacts, consistent with established principles in exposure science and epidemiology, where ambient concentrations—not emission rates—determine inhaled doses and health outcomes.

Consistent with previous research on short-term exposure dynamics, the SdDLCA integrates temporal resolution, local meteorology, population density, and land-use characteristics, providing a refined estimate of human health impacts compared with conventional LCA approaches.

Because concentrations at Site 7 remain systematically below WHO guideline values, the subsequent impact assessment focuses on Site 6, where short-term exposure peaks are most likely to drive measurable health impacts. As shown earlier in Section 3.2, only zones Z1-GS, Z2-FM and Z3-SP exhibit concentrations exceeding WHO thresholds and are therefore considered plausible exposure hotspots.

#### 3.3.1. Daily Spatiotemporal Human Health Impact (DHH(t))

**Figure 9:**
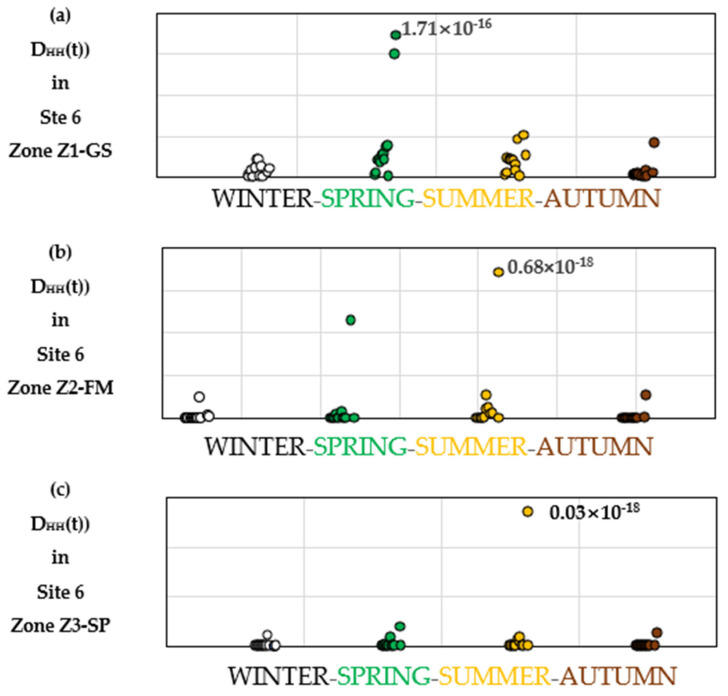
Values of the daily impact on human health, expressed as absolute spatiotemporal damage on human health (D_HH_(t)), resulting from emissions of industrial pollutants studied in zones (**a**) Z1-GS, (**b**) Z2-FM, and (**c**) Z3-SP, near site 6, whose industrial activity consists of all metallurgical operations and where pollutant concentrations exceed WHO thresholds.

Figure 9a–c presents the daily spatiotemporal damage to human health, expressed as DHH(t) using the population density and surface area of the studied zones—Z1-GS, Z2-FM, and Z3-SP—presented in Table 2. The results exhibit marked day-to-day variability, driven by meteorological conditions, emission timing, and the spatial distribution of residents. Peak daily damages are observed in spring for Z1-GS and in summer for Z2-FM and Z3-SP. These patterns reflect local atmospheric behavior: during spring and summer 2020 [39], the region experienced meteorological conditions conducive to atmospheric stability, with above-normal temperatures and a marked deficit in precipitation that limited vertical mixing and favored near-ground accumulation of pollutants within the human breathing zone. According to the national climate summary by Météo-France, summer 2020 was among the warmest on record, with extended dry periods across northern France indicative of persistent anticyclonic conditions that can reduce atmospheric dispersion efficiency.

The coherence between modeled concentrations, meteorological data, and resulting DALY peaks illustrates how SdDLCA captures effectively short-term, site-specific exposure events that would be overlooked by conventional LCA.

#### 3.3.2. Seasonal and Annual Human Health Impact

Daily spatiotemporal damages highlight the importance of high temporal resolution in assessing the impacts of industrial emissions. Seasonal aggregation of these daily values (Figure 9) indicates peak DALY values in spring for Z1-GS and in summer for Z2-FM and Z3-SP, consistent with the daily patterns observed above. These zones, located near Site 6 where metallurgical activities predominate, experienced pollutant concentrations exceeding WHO guideline values for PM_2.5_, NO_x_, and SO_2_.

Local meteorology explains the observed seasonal and spatial variations. Moderate winds (17–22 km/h) would normally facilitate horizontal pollutant transport, but humid conditions and periods of atmospheric stability limited vertical mixing, allowing pollutants to accumulate near the ground within the human breathing zone. Consequently, temporary increases in pollutant concentrations translated into higher DALY values captured by the SdDLCA [40].

Table 2 provides a synthesis of seasonal DALY values, population characteristics, zone area, dominant exposure conditions, and associated meteorological context for zones Z1-GS, Z2-FM, and Z3-SP, reflecting the combined influence of emissions, population distribution, and local meteorology. Detailed meteorological parameters associated with peak exposure periods are reported in Table 3.

These results demonstrate that the SdDLCA framework effectively captures short-term exposure dynamics that would be overlooked by conventional annualized LCA.

### 3.4. Discussion

The results confirm the added value of dynamic, site-specific LCA for assessing human health impacts from industrial air emissions. By explicitly accounting for temporal variability, spatial heterogeneity, and population distribution, the SdDLCA framework enables the identification of exposure hotspots and short-term concentration peaks that are not captured by conventional LCA approaches.

For the production of 497 m^2^ of double-glazed PVC windows, conventional LCA estimates an annual human health impact of 0.13405 DALYs, corresponding to nearly 48 days of healthy life lost per year. For metallurgical processes at Site 6 alone, this impact amounts to 12 days, 17 h, and 48 min annually. These estimates rely on static, aggregated emissions and generic characterization factors, which may overestimate actual exposure-driven health impacts at the local scale.

In contrast, the SdDLCA results indicate extremely low annual impacts, with a maximum value of 1.01 × 10^−15^ DALY for the most exposed zone (Z1-GS). This highlights how site-specific dispersion conditions, population distribution, and temporal emission patterns substantially influence effective exposure and associated health impacts. At the same time, the identification of short-term exposure peaks remains relevant for local air quality management and preventive strategies, even when average annual impacts are low.

## 4. Conclusions

This study demonstrates that Site-dependent Dynamic Life Cycle Assessment (SdDLCA) provides a more realistic evaluation of the human health impacts of industrial air emissions. This approach is illustrated through the production of 497 m^2^ of double-glazed PVC windows for a residential construction project. By introducing spatial and temporal differentiation, the proposed dynamic LCA framework directly links industrial processes to potential health impacts for exposed populations.

Site 6, located in the Hauts-de-France region and hosting metallurgical activities, was progressively identified as the critical site. An initial coupling with the ESPA model allowed the exclusion of sites with negligible emissions. Subsequent atmospheric dispersion modeling enabled a comparison of pollutant concentrations with WHO guideline values, while final coupling with ReCiPe 2016 translated these concentrations into disability-adjusted life years (DALYs), providing a quantitative and interpretable health impact metric.

The results indicate that only areas surrounding Site 6—Z1-GS, Z2-FM, and Z3-SP—exceeded WHO thresholds for PM_2.5_, NO_x_, and SO_2_. Daily and seasonal DALY estimates capture short-term exposure dynamics shaped by emission timing, population distribution, and local meteorological conditions. In comparison, conventional LCA estimates an annual human health impact equivalent to approximately 12 days, 17 h, and 48 min of healthy life lost for emissions from Site 6 alone. The SdDLCA results, by contrast, indicate much lower annual impacts, with a maximum value of 1.01 × 10^−15^ DALY for the most exposed zone, highlighting the influence of site-specific dispersion conditions on effective exposure.

While average annual impacts remain very low, the ability of SdDLCA to identify short-term exposure peaks is particularly relevant for local air quality management and preventive strategies. Future research could extend this framework to secondary pollutants such as ground-level ozone through the integration of advanced dispersion models. Incorporating AI-driven weather forecasts would allow the exploration of future emission and climate scenarios, while accounting for population age distribution could improve the assessment of risks for vulnerable groups such as children and the elderly. These developments would further support evidence-based public health decision-making and environmentally informed industrial planning.

## Figures and Tables

**Table 1:** PMFP values for NO_x_ and SO_2_ emissions and ${CF}_{{PM}_{2.5}}$ value [28,29].

Pollutant	${PMFP}_{s}$ (${kg}_{{PM}_{2.5}}$ $\mathrm{eq}/{kg}_{emitted}$)	${CF}_{{PM}_{2.5}}$ (DALY/kg PM_2.5_)	Perspective *
NO_x_	4.28 × 10^−3^	6.29 × 10^−4^	hierarchist
SO_2_	1.38 × 10^−2^	6.29 × 10^−4^	hierarchist
PM_2.5_	1	6.29 × 10^−4^	-

*: The PMFP values are calculated for a 100-year time horizon and correspond to the hierarchist perspective of ReCiPe 2016, which represents a consensus-based and policy-relevant viewpoint. This perspective relies on broadly accepted scientific assumptions and medium-term time horizons, making it the recommended default for most life cycle impact assessments.

**Table 2:** Population characteristics, seasonal human health impact indicators (DHH(t), DALY), and exposure conditions for the three studied zones surrounding Site 6.

Zone	Number of Inhabitants	Area (m^2^)	Season of Peak Impact	Pollutants Exceeding WHO Thresholds	Typical Meteorological Conditions During Peaks	Seasonal DHH(t) Range (DALY)	Maximum Annual DALY
Z1-GS	23,200	21,440,000	Spring	PM_2.5_, NO_x_, SO_2_	Low wind speed, humid air, atmospheric stability	1.05 × 10^−16^–5.50 × 10^−16^ Days [March–May]	1.01 × 10^−15^
Z2-FM	3533	1,410,000	Summer	PM_2.5_, NO_x_, SO_2_	Moderate winds, stable boundary layer	1.28 × 10^−17^–9.58 × 10^−17^ Days [June–August]	<1 × 10^−15^
Z3-SP	6830	23,400,000	Summer	PM_2.5_, NO_x_, SO_2_	Humid conditions, limited vertical mixing	2.78 × 10^−19^–3.31 × 10^−18^ Days [June–August]	<1 × 10^−15^

**Table 3:** Meteorological conditions during seasonal peaks of human health impacts in zones Z1-GS, Z2-FM, and Z3-SP.

Zone	Dominant Season of Peak Impact	Mean Wind Speed (km/h)	Relative Humidity (%)	Atmospheric Stability	Interpretation for Dispersion
Z1-GS	Spring	17–22	High	Stable	Limited vertical mixing, near-ground accumulation
Z2-FM	Summer	17–22	Moderate–High	Stable	Reduced dispersion efficiency
Z3-SP	Summer	17–22	High	Stable	Pollutant trapping in breathing zone

## Data Availability

The datasets generated during and/or analyzed during the current study are available from the corresponding author on request.

## Abbreviation

The following abbreviations are used in this manuscript:

ESPA	Enhanced Structural Path Analysis
LCA	Life Cycle Assessment
LCI	Life Cycle Inventory
LCIA	Life Cycle Impact Assessment
SdDLCA	Site-Dependent Dynamic Life Cycle Assessment
SDLCI	Spatialized Dynamic Life Cycle Inventory
SdDLCIA	Site-dependent Dynamic Life Cycle Impact Assessment
WHO	World Health Organisation
